# Comparison of Heart Rate Variability in Thai Older Adults with Hypertension, Pre-Hypertension, and Normotension

**DOI:** 10.1155/2024/9631390

**Published:** 2024-05-21

**Authors:** Ruchada Sri-Amad, Nawiya Huipao, Porraporn Sriwannawit, Piyapong Prasertsri, Thapanee Roengrit

**Affiliations:** ^1^Department of Physical Therapy, Faculty of Allied Health Sciences, Chulalongkorn University, Bangkok, Thailand; ^2^Division of Health and Applied Sciences, Faculty of Science, Prince of Songkla University, Songkhla, Thailand; ^3^Department of Physical Therapy, Faculty of Medicine, Prince of Songkla University, Songkhla, Thailand; ^4^Faculty of Allied Health Sciences, Burapha University, Chonburi, Thailand; ^5^Department of Basic Medical Science, Faculty of Medicine Vajira Hospital, Navamindradhiraj University, Bangkok, Thailand

## Abstract

**Objectives:**

This study aims to compare HRV variables across three cohorts: normotensive (NT), prehypertensive (pre-HT), and hypertensive (HT) and to assess the relationship between the blood pressure (BP) and HRV parameters.

**Methods:**

Employing a cross-sectional design, 64 older participants were categorized based on the Joint National Committee's criteria into NT (*n* = 10), pre-HT (*n* = 33), and HT (*n* = 21) groups. Anthropometric data, lipid profiles, and HRV indices were evaluated. HRV data were obtained from the Polar V800 chest strap device using HRV Kubios software for data analysis of short-term recordings lasting 10 minutes. This analysis encompasses both time and frequency domain assessments. The time domain includes the standard deviation of NN intervals (SDNN), the root mean square of successive RR interval differences (RMSSD), and the percentage of successive RR intervals differing by over 50 ms (pNN50). The frequency domain includes low frequency (LF), high frequency (HF), and the ratio of LF-to-HF power (LF/HF). Data were statistically analyzed via one-way analysis of variance (ANOVA) and Pearson correlation.

**Results:**

The HT group exhibited significantly lower values in SDNN, pNN50, LF power, and HF power in comparison to the NT group (*P* < 0.05). Moreover, the HT group had a significantly lower SDNN value compared to the pre-HT group (*P* < 0.05). Inverse associations were uncovered between systolic and diastolic blood pressure and SDNN, pNN50, and HF power (*P* < 0.05). Multiple regression further highlighted the significance of systolic and pulse pressure concerning HF power (*P* < 0.05).

**Conclusions:**

HRV indices are reduced in Thai older adults with HT compared with those with NT. Monitoring HRV in older adults can provide valuable insights into autonomic function and cardiovascular disease risk.

## 1. Introduction

The global population is experiencing a notable surge in the elderly demographics. Projections indicate that by 2030, one-sixth of the world's population will have reached the age of 60 or above, and this figure is set to reach 2.1 billion by 2050. Recognizing that aging is associated with a gradual deterioration in both physical and cognitive capacities, the exploration of health evaluation becomes an essential endeavor within the realms of public health for an aging society.

Hypertension (HT), characterized by BP readings above 140/90 mmHg according to the Eighth Joint National Committee (JNC) [1], is increasingly prevalent worldwide, primarily due to population aging. The adverse effects of HT in older adults are exacerbated by mechanical hemodynamic changes, arterial stiffness, neurohormonal and autonomic dysregulation, and declining renal function [2]. By 2025, it is projected to affect approximately one-third of the world's population [3], contributing to approximately 8.5 million deaths worldwide from stroke, ischemic heart disease, other vascular diseases, and renal disease, as well as pre-HT and other hazardously high BP conditions [4]. Despite its widespread impact, only about 20% of individuals with HT successfully manage their BP. Furthermore, individuals with pre-HT have a 45% higher risk of cardiovascular disease (CVD) events compared to those with normal BP, with an incidence of HT 3.57 times higher in subjects with pre-HT at the baseline than those with normal BP [5].

The autonomic nervous system (ANS) regulates BP through several mechanisms due to its dual function of supplying sympathetic and parasympathetic innervation to bodily organs. Sympathetic nerve endings release noradrenalin, which heightens peripheral vascular resistance, heart rate, and cardiac output [6]. Moreover, the ANS interacts with the renin-angiotensin-aldosterone system, leading to activation that exerts a vasopressor effect, increasing BP by activating the sympathetic nervous system, inhibiting the parasympathetic nervous system, and decreasing baroreflex sensitivity [7, 8]. Impairments in the ANS have been associated with dysfunctional and structural alterations in target organs, increasing the development of various cardiovascular events [6, 7, 9–13].

Heart rate variability (HRV) serves as a noninvasive technique for evaluating the function of the ANS. It has found applications in clinical assessments and monitoring the impacts of therapeutic interventions. HRV is determined by analyzing different parameters that characterize the variability in the time intervals between heartbeats. Activation of the sympathetic nervous system typically results in an increase in HR and a decrease in HRV. Conversely, parasympathetic nervous activity decreases HR while increasing HRV [7].

While an association between HRV and BP has been observed in epidemiological studies, quantifying their relationship remains challenging due to their multifaceted influence on the ANS, such as variations in study populations and measurement techniques. Additionally, previous research indicates impaired parasympathetic drive across different BP levels, as noted in individuals with normal BP, pre-HT, and HT [14]. An autonomic imbalance with increased sympathetic tone is evident, especially in pre-HT but not in HT and normotension.

Therefore, this study aimed to (1) assess the variation in HRV indices within the elderly population across distinct BP subgroups (normal BP, pre-HT, and HT) and (2) explore the associations connecting BP parameters with both time and frequency HRV indices. This information has the potential to establish suitable health monitoring for older individuals susceptible to cardiovascular disease.

## 2. Materials and Methods

### 2.1. Study Population

The cross-sectional study encompassed a cohort of 64 participants. The inclusion criteria for this study were senior Thai individuals aged 60 years and older who were available and willing to participate in this study. Ensuring the health status of seniors is crucial for proactive management. This is particularly important considering their higher risk of certain health conditions and their transition into retirement, which may lead to a potential lack of access to employer-provided health check-ups. Recruitment took place at Hat Yai Chivasuk's Health Promotion Center. The average age of the participants was 67.25 ± 4.53 years. Those with a medical history encompassing cardiovascular disease (CVD), stroke, kidney disease, chronic inflammatory disorders (e.g., psoriasis, rheumatoid arthritis, lupus, and HIV/AIDS), and endocrine diseases (e.g., thyroid diseases and Cushing syndrome) and those currently taking antihyperglycemic, antihypertensive, and lipid-lowering medications were excluded. All participants provided written informed consent, in accordance with the guidelines outlined in the Declaration of Helsinki. The study received approval from the Ethics Committee of the Faculty of Medicine at the Prince of Songkla University (REC 60-166-19-2).

To gather the required data, participants underwent standardized interviews employing a questionnaire that aimed to assess their overall health, current illnesses, and medical history. Pertinent demographic data, such as body weight, height, body mass index (BMI), waist circumference (WC), hip circumference (HC), and waist-to-hip ratio (WHR), were documented for each participant. Measurements for body weight and height were taken, while participants were attired in lightweight clothing and without shoes. BMI was calculated by dividing the body weight (in kg) by the height (in m^2^). A tape measure was employed to gauge WC and HC with precision to the nearest millimeter. WHR was calculated by dividing WC by HC. To ascertain the percentage of body fat, bioelectrical impedance analysis (UM-076 Tanita, Japan) was performed.

### 2.2. Biochemical Analysis

The biochemical analysis aimed to assess participants' health status, specifically evaluating factors such as diabetes mellitus (DM), dyslipidemia, and metabolic syndrome (MetS). The participants' blood samples were collected from the antecubital vein following a 12-hour overnight fasting period. Before the day of the blood collection, all participants were advised to ensure that they had sufficient fasting hours during the same period. Specifically, the participants were advised to keep fasting between 07:00 p.m. and 07:00 a.m. These samples were then placed in a clot activator tube and centrifuged within 1 hour of collection. The lipid profile of the isolated serum (triglycerides (TG), high-density lipoprotein cholesterol (HDL-C), low-density lipoprotein cholesterol (LDL-C), and total cholesterol (TC)) was evaluated through colorimetric enzymatic assays. Furthermore, fasting blood sugar (FBG) was determined using a glucometer (Accu-Chek® Active, Roche Diagnostics GmbH, Mannheim, Germany).

### 2.3. HRV Assessment

This assessment was conducted in an environmentally controlled room (25°C) between 07:00 a.m. and 10:00 a.m. Participants were instructed to restrict the consumption of a heavy meal for 2-3 hours before HRV recording and to refrain from smoking, caffeine, and alcohol for at least 12 hours. Throughout the procedure, participants reclined in a quiet, temperature-controlled room to minimize bodily movement. The skin on the participant's chest was cleaned, following which a chest strap from the HRV monitor (Polar V800™ H7, Electro Oy) was attached over the pectoralis major muscles, utilizing the xiphoid process as a guide. Following a 5-minute rest, HRV data collection occurred over a continuous 10-minute interval. During this time, 600 consecutive RR intervals were extracted for analysis.

The HRV analysis was performed using HRV Kubios software. The time domain variables consisted of the standard deviation of NN intervals (SDNN, an indicator of global autonomic modulation), the root mean square of successive RR interval differences (RMSSD, a marker of vagal modulation), and the percentage of successive RR intervals differing by over 50 ms (pNN50, a measure of parasympathetic activity). Frequency domain variables consisted of low frequency (LF power) (0.04–0.15 Hz, an index of both sympathetic and parasympathetic activity) and high frequency (HF power) (0.15–0.4 Hz, a measure of vagal activity). The ratio of LF-to-HF power (LF/HF, traditionally used to estimate sympathovagal balance) [15] was also included.

### 2.4. Blood Pressure Measurement

Following a 15-minute rest in a sitting position upon arrival at the health center, participants underwent blood pressure measurements between 07:00 a.m. and 10:00 a.m. before moving on to the next experiment. The participant's BP was assessed bilaterally in the upper arms, with the higher value on the corresponding side was recorded. Three measurements with a 5-minute interval were taken, and then the three values were averaged.

Using an automated sphygmomanometer, systolic blood pressure (SBP) and diastolic blood pressure (DBP) were recorded. Pulse pressure (PP) was calculated by subtracting DBP from SBP (PP = SBP − DBP), while mean arterial pressure (MAP) was calculated by adding one-third of the pulse pressure to DBP (MAP = DBP + 1/3 PP).

Using the classification criteria specified in the Joint National Committee's Eighth Report on the Prevention, Detection, Evaluation, and Treatment of High Blood Pressure (JNC8), the participants were categorized into three distinct groups: (1) the HT group with SBP ≥140 mmHg and/or DBP ≥90 mmHg, (2) the pre-HT group with SBP ≥120–139 mmHg and/or DBP ≥80–89 mmHg, and (3) the NT group with SBP <120 mmHg and/or DBP <80 mmHg. The assessment of BP and HRV on the same day not only ensures coherence and reliability within the BP group but also enables the identification of patients with HT as part of our study protocol.

### 2.5. Health Status Classification

Participants' health status was defined according to various criteria: (1) dyslipidemia was identified as TC ≥ 200 mg/dl, TG ≥ 150 mg/dl, LDL-C ≥130 mg/dl, HDL-C <40 mg/dl in men, and HDL-C <50 mg/dl in women [16]. (2) DM was determined as an FBG of ≥126 mg/dl [17]. (3) Obesity was classified using the Asia-Pacific guidelines, using a BMI of ≥25 kg/m^2^ [18]. (4) MetS was defined based on a minimum of three criteria recommended in the revised NCEP ATP III guidelines. The factors considered encompassed increased WC (≥90 cm in men; ≥85 cm in women), increased TC (≥150 mg/dL), low HDL-C (<40 mg/dL in men; <50 mg/dL in women), elevated BP (≥130/85 mm Hg), and elevated FBG (≥100 mg/dL) [19].

### 2.6. Statistical Analysis

The statistical analysis was performed via SPSS software. The normality of the variable distribution was assessed using the Kolmogorov–Smirnov test. Continuous variables were presented as the mean ± standard deviation (SD) if normally distributed or as median (minimum, maximum) if nonnormally distributed. Categorical variables were presented as numbers and percentages. Differences among groups were evaluated using univariate ANOVA or the chi-square test. Pearson's correlation analysis was used to determine the strength and direction of the relationships between BP parameters and HRV indices. Multiple regression analysis was performed to verify the association between HRV indices (independent variables) and BP parameters (dependent variables) at a 95% confidence interval. The threshold for statistical significance was defined as *P* < 0.05.

## 3. Results

A total of 64 Thai elderly individuals (age 67.25 ± 4.53 years) were included in the final data analysis. Among these, 12 participants (18.75%) were male and 52 (81.25) were female. Within the cohort, 10 participants were categorized as healthy controls, 21 had experienced HT, and 33 were part of the pre-HT group. The changes in height, weight, BMI, lipid profile, and the prevalence of dyslipidemia, metabolic syndrome, diabetes, and obesity within the BP subgroups did not demonstrate statistical significance (Table 1). However, the HT group displayed notably higher SBP, DBP, and PP in comparison to the normal BP and pre-HT groups (Table 2).

Concerning time domain variables, the HT group exhibited reduced SDNN and pNN50 compared to the normal BP group (18.73 ± 7.36 ms vs. 33.03 ± 10.92 ms; *P* < 0.05 and 3.98 ± 7.68% vs. 16.28 ± 10.74%; *P* < 0.05, respectively). Moreover, the SDNN value in the HT group was a significantly lower value in SDNN when compared to the pre-HT group (18.73 ± 7.36 ms vs. 22.97 ± 11.01 ms; *P* < 0.05).

**Figure 1:**
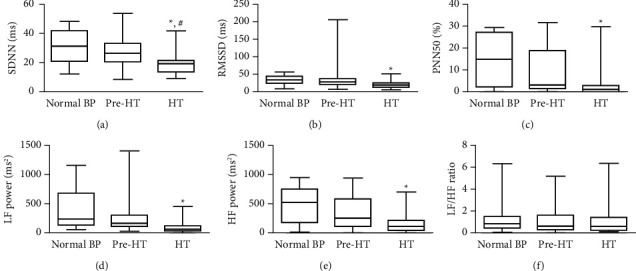
HRV index in the study population grouped by BP classification. Data were presented as mean ± SD. *P* values were derived using the independent *t*-test considering participant characteristic differences in each BP group. ^*∗*^*P* < 0.05 in the HT group versus the normal BP group. ^#^*P* < 0.05 in the HT group versus the pre-HT group. BP, blood pressure; HT, hypertension; SDNN, standard deviation of NN intervals; RMSSD, root mean square of successive RR interval differences; pNN50, percentage of successive RR intervals that differ by more than 50 ms; LF power, low frequency; HF power, high frequency; LF/HF, ratio of LF-to-HF power.

In terms of frequency domain attributes, the HT group demonstrated lower LF power and HF power relative to the normal BP group (107.33 ± 107.50 ms^2^ vs. 480.60 ± 395.75 ms^2^; *P* < 0.05 and 171.38 ± 178.75 ms^2^ vs. 517.10 ± 303.00 ms^2^; *P* < 0.05, respectively). However, there were no significant changes observed in RMSSD and LF/HF (Figure 1).

Pearson's correlation analysis showed a statistically significant negative correlation between SBP and SDNN (*r* = −0.49, *P* < 0.01), pNN50 (*r* = −0.36, *P* < 0.01), LF power (*r* = −0.44, *P* < 0.01), and HF power (*r* = −0.43, *P* < 0.01). Furthermore, a weak yet statistically significant negative association was identified between DBP and SDNN (*r* = −0.35, *P* < 0.01), pNN50 (*r* = −0.29, *P* < 0.02), and HF power (*r* = −0.29, *P* < 0.02). Some HRV metrics (SDNN, pNN50, LF power, and HF power) exhibited associations with PP. A weak correlation between MAP and pNN50 (*r* = −0.27, *P* < 0.03) was observed, while no statistically significant associations emerged between BP parameters and LF/HF ratio and RMSSD (Table 3). Utilizing a multivariate analysis, the relationship between BP parameters and HRV indices was explored. Heightened SBP and PP were associated with decreased HF power (*β* = −0.06, SE = 0.02, *P* < 0.01; *β* = −1.25, SE = 0.02, *P* < 0.01, respectively) (Table 4).

## 4. Discussion

The results of this study indicated that elderly individuals with HT exhibited decreased values in both time domain variables (SDNN, RMSSD, and pNN50) and frequency domain variables (LF power and HF power). This reduced HRV is indeed associated with the progression of age, aligning with an increased likelihood of cardiovascular events. In comparison to individuals with normal BP and those with pre-HT, hypertensive patients exhibit a decline in HRV. Moreover, older adults with HT exhibit lower HRV than older adults with normotension. Additionally, there was a decrease in parasympathetic modulation observed in the hypertensive elderly [20]. Previous studies have revealed an autonomic imbalance characterized by an augmented sympathetic tone, which is more pronounced in the case of pre-HT but not in HT and normotension, particularly in individuals with a family history of HT [14]. Increased BP levels in older adults correspond to a reduced cardiovascular autonomic control. Interestingly, low HRV is indicative of the body experiencing stress, possibly due to impending health problems. However, certain cases in aging populations with bradycardia have demonstrated an increased HRV, suggesting potential cardiac protection attributed to an increased parasympathetic autonomic tone [21].

Generally, the overall sympathetic and parasympathetic activities are indicated by SDNN and LF power. It is recognized that SDNN and LF power are closely related, although SDNN offers greater precision when assessed over a 24-hour period in comparison to shorter periods [15]. Similar results from previous studies also highlight reduced SDNN and LF power in HT patients [22], indicating potential sympathetic hyperactivity and/or impaired parasympathetic tone [23]. A cardiac autonomic profile marked by low SDNN and LF power might denote a 37%–80% increased risk of developing HT. Reduced SDNN among elderly hypertensive patients is associated with a sympathetic overdrive linked to an increased probability of developing HT within 4 years [23]. The phenomenon of sympathetic overactivity appears to be widespread among individuals across various age groups, young, middle-aged, and older, with elevated HT [24]. Furthermore, the arterial baroreflex contributes to the understanding of the relationship between HT and ANS. The baroreceptor reflex, exemplifying parasympathetic activation and sympathetic inhibition, plays a role in short-term BP regulation. Previous studies found a link between lowered HRV and altered baroreflex sensitivity [20], noting associations between cardiac sympathetic tone fluctuations and baroreflex activity in HT patients [25].

Notably, RMSSD, pNN50, and HF power exhibit strong correlations with parasympathetic activity. Enhanced RMSSD aligns with improved cardiovascular health, whereas reduced RMSSD implies lower vagal tone and potential autonomic dysfunction. Consistent with a previous study that demonstrated vagal impairment in the early stages of essential HT [26], both pNN50 and HF power exhibit lower values in hypertensive patients [22]. Moreover, prior studies have highlighted that patients with congestive heart failure exhibited notably reduced pNN50 values than healthy individuals [27].

The current study established a link between higher BP (SBP, DBP, and PP) and decreased HRV (SDNN, pNN50, LF power, and HF power). Elevated SBP is recognized to subject cardiac function to resistance, contributing to myocardial hypertrophy. Consequently, high BP poses detrimental effects on cardiac health [28]. In women, an inverse relationship was observed between both SBP and DBP and HRV, whereas no such connection was found between SBP and HRV in men [6]. Moreover, SBP had a negative correlation with SDNN, RMSSD, and HF power exclusively among female patients with uncomplicated arterial HT. However, a decrease in DBP was linked to increased SDNN [9], RMSSD, LF power, and HF power in both genders [29]. Given that women showed a higher vagal activity index in HRV compared with men [30], the impact of sex differences on baroreceptor reflex sensitivity and HRV should be acknowledged. Moreover, autonomic function parameters were especially impaired in hypertensive women compared with hypertensive men [22]. Our findings contrast with those of another study, wherein individuals under 65 years displayed a negative correlation between MAP, RMSSD, and HF power [31]. Notably, elevated home MAP during morning BP measurements, in contrast to evening measurements, was significantly associated with low RMSSD and HF power. Moreover, DBP exhibited an inverse relationship with RMSSD during morning BP measurements [31], suggesting ANS activity's dependence on circadian variations. However, variations in HRV are influenced by multiple factors, including physiological, neuropsychological, pathological, and environmental elements, contributing to the diverse and disputed outcomes concerning the correlation between HRV and BP [32].

The study's limitations warrant attention. First, the participant pool was limited in size. The results show a lack of conformity among the groups. This may primarily be attributed to three factors: the mean difference potentially being too small, the standard deviation potentially being too large, and the sample size possibly being insufficient, especially within the normal BP group. Moreover, the study followed a cross-sectional and community-based approach, thus lacking national representation. A broader participant base would require further examination for a comprehensive analysis. Second, the study utilized a single HRV assessment with short-term recordings, and BP measurements were exclusively conducted in the morning. Previous studies have highlighted the influence of circadian fluctuations on ANS activity. Additionally, participants who experienced nighttime nondipping were not excluded. Although this condition may result in a higher 24-hour mean BP level, this study tried to construct similar conditions that could potentially influence BP for all participants. For example, BP assessments were performed at the same time of day (between 07:00 a.m. and 10:00 a.m.) and under similar environmental and internal conditions (e.g., room temperature, humidity, and sounds). The participants were advised to ensure they got enough sleep before the day of the experiments; they were also instructed to rest in a sitting position for 15 minutes before BP measurement and to relax while measuring BP.

## 5. Conclusion

Thai older individuals with HT had lower SDNN, pNN50, LF power, and HF power values compared with those with NT. These data suggest reduced parasympathetic activity and baroreceptor reflex responses in HT. Monitoring HRV in older adults can provide valuable insights into autonomic function and cardiovascular risk, and interventions aimed at improving HRV may have potential benefits for preventing cardiovascular disease [20, 33].

## Figures and Tables

**Table 1:** Participant characteristics divided by the BP group.

Characteristics	Normal BP (*N* = 10)	Pre-HT (*N* = 33)	HT (*N* = 21)	*P* values
Physical characteristics				
Age (years)	64.40 ± 4.33	67.03 ± 4.61	68.95 ± 3.85	ns
Male, no. (%)	0 (0)	8 (24.24)	4 (19.04)	ns
Height (cm)	153.5 (148, 160)	157 (140, 180)	155 (137, 172)	ns
Weight (kg)	60.05 ± 7.77	59.51 ± 6.68	59.50 ± 7.14	ns
BMI (kg/m^2^)	25 (19.65, 32.03)	23.83 (20.07, 33.16)	23.66 (18.73, 36.23)	ns
WC (cm)	89 (73, 104)	83 (71, 98)	83 (73, 108)	ns
HC (cm)	98.60 ± 4.94	96.91 ± 4.44	97.81 ± 7.10	ns
WHR (cm)	0.88 (0.77, 1.05)	0.87 (0.80, 0.99)	0.85 (0.77, 1.04)	ns
Body fat (%)	35.30 ± 4.57	32.62 ± 4.64	31.36 ± 5.40	ns
Resting HR (beats/min)	70.70 ± 7.86	67.55 ± 9.49	71.57 ± 9.03	ns
Biochemical parameters				
TC (mg/dL)	228.10 ± 47.20	241.12 ± 43.64	227.19 ± 51.40	ns
HDL-C (mg/dL)	63 (43, 100)	63 (45, 110)	58 (34, 101)	ns
TG (mg/dL)	93 (54, 214)	81 (37, 247)	86 (41, 268)	ns
LDL-C (mg/dL)	149.78 ± 42.15	166.40 ± 42.11	150.30 ± 47.47	ns
FBS (mg/dL)	94 (82, 109)	98 (89, 243)	102 (89, 155)	ns
Health status				
Dyslipidemia, no. (%)	8 (80)	27 (81.81)	18 (85.71)	ns
DM, no. (%)	0 (0)	1 (3)	3 (14.28)	ns
MetS, no. (%)	1 (10)	3 (9.09)	7 (33.33)	ns
Obese, no. (%)	6 (60)	8 (24.24)	7 (33.33)	ns

Continuous variables were presented as the mean ± SD or median (minimum, maximum). Categorical variables were presented as numbers and percentages. *P* values were derived using the ANOVA or chi-square test. HT, hypertension; BMI, body mass index; HC, hip circumference; WHR, waist-to-hip ratios; HR, heart rate; TC, total cholesterol; HDL-C, high-density lipoprotein cholesterol; TG, triglyceride; LDL-C, low-density lipoprotein cholesterol; DM, diabetes mellitus; MetS, metabolic syndrome; ns, no significance.

**Table 2:** BP parameters and HRV index in the study population grouped by BP classification.

	Normal BP (*N* = 10)	Pre-HT (*N* = 33)	HT (*N* = 21)
BP parameters			
SBP (mmHg)	113 ± 5.77	128 ± 6.33	153 ± 11.87^*∗*#^
DBP (mmHg)	71 ± 4.77	78 ± 5.20	86 ± 8.02^*∗*#^
PP (mmHg)	41 (35, 50)	50 (32, 59)	64 (52, 100)^*∗*#^
MAP (mmHg)	86 (75.33, 88.67)	86 (86, 108)	91 (86, 125)^*∗*^
Time domain			
SDNN (ms)	33.03 ± 10.92	26.97 ± 11.01	18.73 ± 7.36^*∗*#^
RMSSD (ms)	34.29 ± 13.92	34.57 ± 33.16	20.89 ± 10.42^*∗*^
pNN50 (%)	16.28 ± 10.74	8.90 ± 10.21	3.98 ± 7.68^*∗*^
Frequency domain			
LF power (ms^2^)	480.60 ± 395.75	271.67 ± 317.48	107.33 ± 107.50^*∗*^
HF power (ms^2^)	517.10 ± 303.00	330.45 ± 249.94	171.38 ± 178.75^*∗*^
LF/HF ratio	1.67 ± 1.95	1.17 ± 1.23	1.25 ± 1.55

Data were presented as the mean ± SD. *P* values were derived using the independent *t*-test considering participant characteristic differences in each BP group. ^*∗*^*P* < 0.05 in the HT group versus the normal BP group. ^#^*P* < 0.05 in the HT group versus the pre-HT group. BP, blood pressure; HT, hypertension; systolic blood pressure; DBP, diastolic blood pressure; PP, pulse pressure; MAP, mean arterial pressure; SDNN, standard deviation of NN intervals; RMSSD, root mean square of successive RR interval differences; pNN50, percentage of successive RR intervals that differ by more than 50 ms; LF power, low frequency; HF power, high frequency; LF/HF, ratio of LF-to-HF power.

**Table 3:** Correlation of the HRV index with BP parameters of the study population.

	SBP		DBP		PP		MAP	
	*r*	*P* value	*R*	*P* value	*r*	*P* value	*r*	*P* value
Time domain								
SDNN (ms)	−0.49	<0.01^*∗∗*^	−0.35	<0.01^*∗∗*^	−0.42	<0.01^*∗∗*^	−0.24	0.05
RMSSD (ms)	−0.25	0.05	−0.22	0.08	−0.19	0.13	−0.23	0.07
pNN50 (%)	−0.36	<0.01^*∗∗*^	−0.29	0.02^*∗*^	−0.29	0.02^*∗*^	−0.27	0.03^*∗*^
Frequency domain								
LF power (ms^2^)	−0.44	<0.01^*∗∗*^	−0.20	0.12	−0.46	<0.01^*∗∗*^	−0.10	0.45
HF power (ms^2^)	−0.43	<0.01^*∗∗*^	−0.29	0.02∗	−0.38	<0.01^*∗∗*^	−0.23	0.07
LF/HF ratio	−0.05	0.72	0.08	0.51	−0.11	0.37	0.10	0.42

Analysis was performed using Pearson's correlation analysis. ^*∗*^*P* < 0.05, ^*∗∗*^*P* < 0.01. BP, blood pressure; SBP, systolic blood pressure; DBP, diastolic blood pressure; PP, pulse pressure; MAP, mean arterial pressure; SDNN, standard deviation of NN intervals; RMSSD, root mean square of successive RR interval differences; pNN50, percentage of successive RR intervals that differ by more than 50 ms; LF power, low frequency; HF power, high frequency; LF/HF, ratio of LF-to-HF power.

**Table 4:** Multiple regression analysis for BP parameters and related HRV indices among participants.

	Dependent variable												
	SBP			DBP			PP				MAP		
	*β*	SE	*P* value	*β*	SE	*P* value	*β*		SE	*P* value	*β*	SE	*P* value
Time domain													
SDNN (ms)	−0.53	0.39	0.18	−0.35	0.20	0.21	−0.24		0.31	0.37	−0.14	0.24	0.63
RMSSD	−0.01	0.08	0.88	−0.08	0.04	0.54	0.02		0.06	0.83	−0.18	0.05	0.21
pNN50 (%)	1.37	0.61	0.12	0.09	0.31	0.81	1.05		0.47	0.11	−0.49	0.37	0.23
Frequency domain													
LF power (ms^2^)	0.00	0.01	0.63	0.15	0.01	0.49	0.03		0.01	0.88	0.14	0.01	0.53
HF power (ms^2^)	−0.06	0.02	0.01^*∗*^	−0.26	0.01	0.55	−1.25		0.02	0.01^*∗*^	0.31	0.01	0.49
LF/HF ratio	−2.99	1.71	0.08	−0.01	0.88	0.92	−0.33		1.32	0.05	0.03	1.05	0.82
*R*-squared	0.297			0.192				0.518			0.137		
Adjusted *R*-squared	0.223			0.106				0.191			0.047		

Analysis was performed using a multiple regression method. ^*∗*^*P* < 0.05. *β*, standardized beta; SE, standard error; SBP, systolic blood pressure; DBP, diastolic blood pressure SDNN, standard deviation of NN intervals; pNN50, percentage of successive RR intervals that differ by more than 50 ms; LF power, low frequency; HF power, high frequency.

## Data Availability

The data used to support the findings of this study are available from the corresponding author upon request.
